# Contact Allergy: A Review of Current Problems from a Clinical Perspective

**DOI:** 10.3390/ijerph15061108

**Published:** 2018-05-29

**Authors:** Wolfgang Uter, Thomas Werfel, Ian R. White, Jeanne D. Johansen

**Affiliations:** 1 Department of Medical Informatics, Biometry and Epidemiology, University of Erlangen/Nürnberg, 91054 Erlangen, Germany; 2 Division of Immunodermatology and Allergy Research, Department of Dermatology and Allergy, Hannover Medical School, 30625 Hannover, Germany; Werfel.Thomas@mh-hannover.de; 3 St John’s Institute of Dermatology, Guy’s Hospital, London SE1 9RT, UK; ian.white@kcl.ac.uk; 4 Department of Dermatology and Allergy, National Allergy Research Centre, Copenhagen University Hospital Herlev and Gentofte, 2900 Hellerup, Denmark; Jeanne.Duus.Johansen@regionh.dk

**Keywords:** allergic contact dermatitis, contact allergy, exposure, review

## Abstract

Contact allergy is common, affecting 27% of the general population in Europe. Original publications, including case reports, published since 2016 (inclusive) were identified with the aim of collating a full review of current problems in the field. To this end, a literature search employing methods of systematic reviewing was performed in the Medline^®^ and Web of Science™ databases on 28 January 2018, using the search terms (“contact sensitization” or “contact allergy”). Of 446 non-duplicate publications identified by above search, 147 were excluded based on scrutiny of title, abstract and key words. Of the remaining 299 examined in full text, 291 were deemed appropriate for inclusion, and main findings were summarised in topic sections. In conclusion, diverse sources of exposures to chemicals of widely-differing types and structures, continue to induce sensitisation in man and may result in allergic contact dermatitis. Many of the chemicals are “evergreen” but others are “newcomers”. Vigilance and proper investigation (patch testing) are required to detect and inform of the presence of these haptens to which our populations remain exposed.

## 1. Introduction

Contact allergy (synonymous term “contact sensitization”) is a common form of delayed type hypersensitivity, usually to small contact allergens (haptens) <1000 Da, but sometimes also to larger molecules. A hapten is a molecule that can elicit an immune response only when attached to a carrier molecule, mostly skin self-proteins; from a clinical perspective, the term contact allergen is preferred and used here. Following sensitization, a subsequent exposure above the individual elicitation threshold will result in clinically visible disease, namely, allergic contact dermatitis (ACD) or, more rarely, mucositis. The diagnosis of contact allergy is made with the patch test. Although this test is more than 100 years old, it is still the standard diagnostic approach which has achieved a high degree of standardization. A European guideline has recently been published giving a complete yet concise description of the background, indication, technique, and interpretation of the patch test [1].

Contact allergy is common: according to a large epidemiological study, 27% of the representative sample of the general population in 5 European countries had positive reactions to at least one of the contact allergens included in a so-called “baseline series” (previously called “standard series”) [2]. In clinical use, a baseline series is applied to every patient being patch tested, as it comprises those contact allergens which are generally considered most important. Usually, the baseline series, a set of 30 contact allergens [3], is supplemented with special test series of commercial contact allergens tailored to individual exposures (e.g., hair cosmetics, rubber, resins, metals) to increase the likelihood of a positive, clinically relevant result beyond the screening offered by the baseline series.

However, although several hundred contact allergens are available as commercially-prepared patch test preparations from different manufacturers world-wide, there are two aspects indicating that even this broad array may not be fully sufficient for achieving a diagnosis in all patients: (i) the availability of commercial patch test contact allergens for use in clinical practice may be limited, e.g., due to restrictions in licensing these [4] and (ii) there are many more substances which have actually been reported to be contact allergens in case reports or small series, and the scope of these is ever-expanding. To avoid a diagnostic gap, this calls both for inclusion of patient’s own products in the patch test, and for constantly updated awareness of “new” contact allergens, or “old” contact allergens encountered in new exposure contexts. The present review aims at addressing the latter aspect by providing a structured overview of important new evidence which has accumulated in the last 2 years. By incorporating methods used in systematic reviews, namely, a systematic approach to literature retrieval, we have tried to identify all pertinent publications. 

## 2. Methods

The objective of the present review is to give a full overview of evidence concerning aspects of contact allergy important for the clinician. Therefore, results of basic immunological or chemical studies or on treatment of ACD are not included. Neither were other reviews or commentaries, as the present review should be based on original reports only except in the discussion part, where appropriate and important. Moreover, cutaneous adverse drug reactions (CADR) are excluded, except if a manifestation of systemic allergic dermatitis (SAD; note that this is the preferred term for what is sometimes called “systemic allergic contact dermatitis”, which is incorrect), i.e., previous sensitization by topical exposure of the skin, and then the development of allergic dermatitis via systemic exposure. Contact urticaria and protein contact dermatitis or other IgE-mediated disease was similarly disregarded, as was the special topic of photosensitivity. Conversely, publications addressing exposure analysis and deemed important for the management of contact allergic patient are included. 

As the search phrase, (“contact sensitization” OR “contact allergy”) AND (“2016/01/01” [Date—Publication]: “3000” [Date—Publication]), 3000 meaning until the current day, was used. A search of the Medline^®^ database using above search string was performed on 28 January 2018. In addition, Web of Science™ (WoS) was used, employing the same search terms. Both search results were merged and duplicates removed. The results were analysed on the level of title and abstract and either excluded, if not pertinent to the research question, or further processed by reviewing the full text. The process was performed by a single investigator (W.U.), as duplicate/consensualised extraction of information and, particularly, assessment of bias of reports normally employed in the preparation of systematic reviews was not deemed necessary in the present strictly descriptive context. The main aim was to sensitively identify all pertinent publications.

The initial search directly at the PubMed portal yielded 308 references. A search performed within 1 h on a Sunday at WoS identified altogether 410 references, of these, 390 in the “Science Core Collection”, 316 in “Current Contents Connect”, 3 in SciELO Citation Index, interestingly 357 in Medline^®^ and, moreover, 169 in BIOSIS Citation Index—evidently with substantial overlap. When merging the search data sets between PubMed and WoS™, 131 records were identified only via WoS™ and 35 only via PubMed, while 279 were identified via both portals. Of note, meeting abstracts are not included in Medline^®^ but in WoS, which explains a large part of the discrepancy and asymmetry. In total, 445 non-duplicate records were identified.

These 445 publications were screened on the level of title, abstract, and key words, leading to the exclusion of altogether 147 publications, due to the following reasons (in descending order): review (*n* = 72), basic immunology or chemistry (*n* = 32), not concerning contact allergy (*n* = 38), cutaneous adverse drug reaction (*n* = 2) and contact urticaria (*n* = 3). Hence, 298 publications were assessed based on the full text. This assessment led to further exclusions for the following reasons: meeting abstract only (*n* = 20), basic immunology/chemistry/genetics (*n* = 13), review (*n* = 10), CADR (*n* = 1), duplicate publication (*n* = 1), internal disease (*n* = 3), other dermatitis (*n* = 1), only available as pre-print (*n* = 1), photo-contact allergy [1]. Furthermore, in 16 cases, the publications were regarded as either definitely “not novel” or with severe methodological flaws and thus not eligible for inclusion. The final set of references thus identified was supplemented by manual search results in the two main subspecialty journals in the field, “Contact Dermatitis” and “Dermatitis”. Thereby, 291 publications were eventually included in this review, including 3 reviews and guideline papers partly not falling into the search period.

## 3. Results

The information contributed by the included studies is presented in topical subsections. The inclusion in one or the other subsection may be partly arbitrary but is hoped to enable a quick overview.

### 3.1. Contact Allergy in the Population

The BAMSE population-based birth cohort study involved patch testing at the 16-year follow-up. Of the 2285 patch tested participants, 15.3% had at least one positive reaction, more commonly girls than boys (17.0% versus 13.4%). The most common contact allergens were nickel (7.5%), fragrance mix (FM) I (2.1%), and *p*-*tert*-butylphenol formaldehyde resin (1.9%) [5]. In the same study, exposures possibly leading to sensitisation have been addressed: piercing was self-reported by 55.4% and hair dyeing by 50.1%, especially girls. Significant associations between piercing and symptoms of metal intolerance, respectively, and nickel allergy (odds ratio (OR) 1.77, 95% confidence interval (CI): 1.04–3.03 and 2.25, 95% CI 1.57–3.23, respectively, were found). Skin symptoms after use of make-up and personal hygiene products was associated with positive reactions to FM I (OR 2.11, 95% CI: 1.02–4.35) [6].

A cross-sectional study of a random sample from the general population, aged 18–74 years, in five different European countries (Sweden, The Netherlands, Germany, Italy, and Portugal) included 3119 participants who were patch tested with TRUE Test^®^ panels 1–3 and FM II, hydroxyisohexyl 3-cyclohexene carboxaldehyde (HICC) and sesquiterpene lactone mix. In this sample, 27.0% had at least one positive reaction to above set of contact allergens, most often to nickel (14.5%), thiomersal (5.0%), cobalt (2.2%), FM II (1.9%), FM I (1.8%), hydroxyisohexyl 3-cyclohexene carboxaldehyde (HICC, a component of FM II; 1.4%), and *p-tert*-butylphenol formaldehyde resin (1.3%). Interestingly, the risk of being sensitised was not increased in those with atopic dermatitis (prevalence 7.6%, odds ratio 1.0). In view of more than one-quarter of the general European population being allergic, improvements of primary prevention of contact allergy need to be enforced, according to the authors [2]. 

It may generally be argued that any diagnostic test with a less-than-perfect sensitivity and particularly specificity will yield a positive predictive value which is far from 100%, i.e., a certain proportion of false-positive results [7]. The extent cannot actually be estimated, as data on the “true prevalence” of sensitization (in the population) are lacking—given the fact that the patch test itself is the standard tool for diagnosing contact sensitization. Further studies using the repeated open application test (ROAT) as verification are aiming at providing more evidence on this particular aspect.

The consequences of being contact allergic have been assessed in a Danish study with 2270 participants from a population-based study followed up ~5 years after a patch test; even though a history of atopic dermatitis was the strongest risk factor for persistent or incident hand dermatitis (OR 9.0), filaggrin (*FLG*) null mutation (OR 3.0), and contact sensitisation (OR 2.5) were also independently associated with persistent hand dermatitis [8]. Conversely, as has been pointed out based on a study in 1828 patients, constitutional impairment of the epidermal barrier (such as *FLG* null mutation) or acquired barrier defects e.g., owing to wet work may promote sensitization not only to strong, but also to weak contact allergens [9]. 

### 3.2. Patch Testing with the Baseline Series

From the North American Contact Dermatitis Group patch test results from 2013/14, 4871 patients were reported. About 2/3 had at least one positive reaction (to a baseline series with 70 contact allergens, i.e., twice as extensive as the European baseline series). In case of 2 contact allergens, sensitisation prevalence had increased significantly, namely, methylchloroisothiazolinone/methylisothiazolinone (MCI/MI) and 2-hydroxyethyl methacrylate. Similar to findings from Europe, methylisothiazolinone caused 10.9% positive reactions, being the third most common contact allergen [10]. 

An indirect assessment of contact allergy prevalence in a larger set of patients tested with a baseline series has been used in North America by analysing usage of the Contact Allergy Management Program, a database including some 5000 topical products, in terms of how often for certain substances information had been retrieved. While being an interesting approach of secondary data analysis, this is not entirely devoid of some biases also discussed by the authors, mostly the focus on topical products which leads to an under-representation of contact allergens encountered in other types of products [11]. Interestingly, the same database allows for the estimation of usage of contact allergens in topical/cosmetic products, and thus can serve as denominator information [12]. 

### 3.3. Contact Allergy in Children and in Atopic Patients

A retrospective analysis of de-identified patch test results of children aged 18 years or younger, entered by participating providers in the Pediatric Contact Dermatitis Registry in the USA, during the first year of data collection (2015–2016) presented results on 1142 cases from 34 US states, entered by 84 providers. Sixty-five percent of cases had one or more positive patch test reaction, and 48% 1 or more relevant positive patch test. Positive reactions most common to nickel (22%), FM I (11%), cobalt (9.1%), *Myroxylon pereirae* (balsam of Peru; 8.4%), neomycin sulfate (7.2%), propylene glycol (6.8%), cocamidopropyl betaine (6.4%; false-positive, irritant reactions possible), bacitracin (6.2%), formaldehyde (5.7%), and gold (5.7%) [13]. 

Between 2005 and 2014, among 500 consecutive children aged ≤16 patch tested at Leeds Teaching Hospitals were identified, 27% had one or more positive patch test findings. Allergy to nickel was the most frequent finding in 18%, followed by FM I (17%), *p*-phenylenediamine (16%), and MCI/MI (6%). Decreases in the observed prevalences were attributed to changes in European legislation and cosmetic product use in children [14]. In a bi-centric Dutch study, 1012 children and adolescents were tested for suspected contact allergy, and almost half of them had at least one positive reaction. In children with atopic dermatitis (*n* = 526) sensitisation to lanolin alcohol, Amerchol™ L 101 (a lanolin product), FM I and *Myroxylon pereirae* (balsam of Peru) was significantly more common than in non-atopic patients [15]. 

In Padova, Italy, 2614 children younger than 11 years were patch tested with a baseline series of 30 contact allergens, and 1220 children (46.7%, thus similar to above-mentioned Dutch study) developed at least one positive reaction, 606 of which were clinically relevant (49.7%). The most frequent reactions were to nickel sulfate (22.7%), cobalt chloride (11.1%), potassium dichromate (9.9%), neomycin sulfate (5.2%), thiomersal (4.2%), cocamidopropyl betaine (3.4%), and methylchloroisothiazolinone/methylisothiazolinone (3.2%), similar in children with and without atopic dermatitis [16]. Among 109 Iranian children and adolescents patch tested 2007–2009, 46.8% had at least one positive reaction, most often to nickel (19.3%), cobalt (10.1%), and methylisothiazolinone (6.4%) [17]. 

In a cross-sectional Danish study, 100 children and adolescents aged 5–17 years with a diagnosis of atopic dermatitis were patch tested with a paediatric series of 31 contact allergens. In total, 30% had at least one positive patch test reaction, in 17/100 this was relevant to the current skin symptoms. The risk of contact allergy was significantly correlated to the severity of atopic dermatitis. Metals and components of topical skincare products were the most frequent sensitizers. Therefore, the need for patch testing children with atopic dermatitis is confirmed, as they may have unrecognised contact allergies contributing to, or maintaining, their skin symptoms [18]. 

A case report from Estonia illustrates the common observation that treatment-related contact allergy and ACD may complicate chronic atopic eczema; in the present case sesquiterpene lactones from chamomile wet dressings had been identified as one of the culprits [19].

### 3.4. Metals

In consecutive patients patch tested in 12 European countries within the ESSCA (www.essca-dc.org) network, 19.0% were positive to nickel sulfate, 6.6% to cobalt chloride and 4.3% to potassium dichromate, with partly striking differences between some countries; notably, nickel and chromium contact allergy prevalences were particularly low in Denmark with a long-standing regulation of these contact allergens [20]. 

#### 3.4.1. Nickel

In some European countries, and for more than a decade in the European Union (EU) as a whole, nickel has been regulated in terms of limiting the release to 0.5 µg/cm^2^/week in objects with close contact with the skin, and 0.2 µg/cm^2^/week in piercing posts—notwithstanding some shortcomings of this regulation, reviewed in Ref. [21]. The decisive role of piercing was confirmed in a hitherto unregulated country, the US, in a retrospective cross-sectional analysis of 17,912 patients patch tested from 2007 to 2014. In this study, nickel sensitivity was associated with 1 or more piercing for men and women combined (relative risk (RR), 2.54; 95% CI: 2.35–2.75), with risk in increasing with number of piercings [22]. 

In 2014, the definition of nickel contact potentially relevant for elicitation of ACD under REACH (Registration, Evaluation, Authorisation and Restriction of Chemicals) was re-defined in the EU by extending the concept of “prolonged contact” (typically with e.g., earrings etc.) to repeated short contacts either >10 min on three or more occasions or >30 min on one or more occasions within a 2-week period. Indeed, according to a Danish study on current self-reported metallic exposures leading to dermatitis in nickel-allergic patients and the minimum contact time needed for dermatitis to occur, 21.4% of the patients reported dermatitis reactions within 10 min of contact, and dermatitis reactions within 30 min of contact were reported by 30.7% of patients [23]. In other regions of the world, such as Kuwait, nickel is a very important contact allergen, with 40% positive reactions in 2461 consecutively patch tested patients; the authors recommend adoption of nickel regulation limiting exposure [24]. 

A Swedish survey tested 141 accessories, utensils for needlework, painting, and writing and electronic devices with the dimethylglyoxime (DMG) test and found 44% positive (i.e., releasing nickel), while 9% gave a doubtful test result [25]. Also, some surfaces of some laptop computers potentially coming into contact with the skin have been found to release nickel [26]. Oral/intestinal exposure to sufficient doses of nickel ions may trigger systemic allergic dermatitis, with large inter-individual variation related to the elicitation threshold. Italian researchers examined nickel liberation from new and used cooking utensils under realistic conditions and found a limited release, which, combined with nickel intake from foods themselves, could exceed the elicitation threshold in some sensitised subjects [27]. Actual skin deposition after playing with children’s toys with known nickel release for 30 s was identified by the DMG test on the exposed skin in 2 of the 3 toys [28].

The reproducibility of patch testing with nickel using a highly standardised test system, the TRUE Test^®^, was investigated in a small series of 819 patients tested at least twice in Odense, Denmark. Reproducibility of nickel was 67%, followed by chromium (62%) and cobalt (61%) [29]. The lymphocyte transformation test (LTT) is being used for the diagnosis of contact sensitisation and has proven useful in some particular circumstances. Using optimised stimulation conditions, Ständer et al., based on results of 50 controls and 50 subjects with self-reported “nickel allergy” have found the LTT a useful alternative to patch testing [30], e.g., if the skin condition would not permit patch testing.

#### 3.4.2. Chromium

Exposure to chromium has been regulated in cement, and recently in leather in the EU. Concerning the former, this infamous cause of cement dermatitis has lost much of its significance. However, isolated cases may still be identified, as illustrated by the results of an investigation of 24 workers in a plant producing pre-cast concrete elements, where 4 workers were found sensitised to dichromate (as well as to ethylene diamine which had been added to the concrete) [31]. An explanation of sensitisation could be that the reduction of chromium offered by the added ferrous sulfate is reversible, e.g., by air exposure and subsequent autoxidation. 

Concerning leather exposure, to provide insights for risk assessment and patient counselling, Danish authors examined whether the handling of chromium-containing samples of leather and metal results in the deposition of chromium onto the skin in a small sample of 5 healthy volunteers, who handled samples of leather and metal known to contain and release chromium for 30 min. Acid wipe sampling of the participants’ fingers showed chromium deposition after exposure to leather (range 0.01–0.20 µg/cm^2^) in all, handling metal discs (range 0.02–0.04 µg/cm^2^) in 3/5 participants, i.e., significant levels, in spite of a short duration of exposure [32]. 

A spot test for the semi-quantitative detection of chromium-VI (dichromate) based on diphenylcarbazide is available since some years ago. With this, no release of dichromate was detected in 848 jewellery items, of which 19% had been previously shown to contain chromium (oxidation state not determined) [33]. However, patch testing chromium-coated disks (a surface treatment often found in metal parts such as screws, bolds, nuts, and washers) in 10 chromium-allergic patients (compared to 5 non-sensitised controls) demonstrated that at least some of the samples elicited positive patch test reactions in up to 4/10 patients [34].

#### 3.4.3. Cobalt

Cobalt contact allergy has indiscriminately been attributed to concomitant nickel sensitisation, either due to immunological cross-reactivity (which has not been actually demonstrated experimentally) or coupled exposure (which may be over-estimated). A Swedish study in 656 consecutive patients found positive patch tests to cobalt chloride 1% pet. in 14%; half of these did not react to nickel, illustrating independent sensitisation [35].

Dental prostheses made (partly) from cobalt-containing metal alloys may contribute to oral mucosal cobalt exposure while new; however, used prostheses were found to be passivated and did not release any cobalt [36]. As some pigments are salts of cobalt, exposure via these may cause ACD. This has been established as likely cause of periorbital ACD in 2 Korean females, whose eye shadows contained 6.8–120 mg/kg Co, corresponding to calculated skin doses of 0.01–0.1 µg/cm^2^ [37]. 

In accordance with previous reports, 2 Danish patients (one 12-year-old boy and a 70-year-old man) developed severe, partly wide-spread ACD due to cobalt exposure in a leather cushion and a new leather sofa, respectively, shown to contain 802 ppm and 1250 ppm cobalt by inductively coupled mass spectrometry [38]. 

#### 3.4.4. Other Metals

A 5-year-old boy who loved to play with coins and various toys developed fingertip dermatitis, which was attributed to copper release from some materials, and subsequent sensitisation detected by patch testing with copper(II) sulfate pentahydrate 2% pet. [39]. A malachite necklace caused eczema in a 63-year-old patient, which could be attributed to sensitisation to copper (copper sulfate 2% pet.: +, copper oxide 5% pet.: ++), which is a major constituent of this semi-precious mineral [40].

Aluminium contact sensitisation has gained some attention due to the observation of cutaneous/subcutaneous inflammation after injection of aluminium absorbed vaccines [41,42]. A Danish series investigated 47 children with subcutaneous nodules caused by childhood vaccinations 2003 to 2013. Most patients had a typical presentation of persisting pruritic subcutaneous nodules, and 42 had a patch-test performed with aluminium chloride hexahydrate 2% pet., positive in 39 (92%). The persistent skin reactions were treated with potent topical corticosteroids and disappeared slowly. Although families were advised to continue vaccination of their children, one-third of parents omitted or postponed further vaccinations [43]. Patch test diagnosis can employ either aluminium lactate or aluminium chloride hexahydrate; the most appropriate test preparation is still being discussed. With both salts in various concentrations, Siemund et al. noted a high variability over time upon re-testing; therefore, false-negative reactions are possible and repeat testing is recommended in case of strong suspicion of aluminium contact allergy [44]. A 50-year-old Spanish patient was patch tested to various metals before placement of a metal dental implant. While the patch test was positive to nickel, cobalt, and gold sodium thiosulfate until D7, additional positive reactions appeared around D20; upon re-testing, additional positive reaction to cadmium, indium, and tin were noted, and thus active sensitisation was diagnosed [45]. 

Titanium is largely regarded as a weak sensitiser; moreover, patch testing with titanium is not yet well standardised. In a patient with extensive, long-standing tattoos, ACD developed some time after implantation of titanium metal in the course of spinal surgery, accompanied by symptoms of general malaise. Both disappeared 1 week after removal of the implants. While patch test results with different titanium preparations remained inconclusive, with a single ++ reaction to titanium 10% pet. on day 4 at the arm, but not on the back, the clinical course, and detection of titanium, amongst other trace metals, in the tattoos made delayed-type sensitisation to titanium very likely [46]. 

Vanadium is a metal included in a variety of corrosion-resistant alloys. In two US patients who underwent spinal surgery with vanadium-containing material, dermatitis over the areas of paravertebral implant developed. Patch testing with metallic vanadium (concentration not given) and in one case also with vanadium pentoxide 10% pet. was positive, and dermatitis (slowly) resolved after the removal of the implants, supporting the diagnosis [47]. 

The clinical significance of positive patch test reactions to metals in general, and to gold salts in particular, is much debated; a recent analysis of patch test reactions to gold sodium thiosulfate included in the TRUE Test^®^ in 35/149 positively reacting patients in Tenerife, Canary Islands demonstrated lacking clinical relevance in virtually all of these, prompting the authors to recommend exclusion of this contact allergen from routine testing [48].

Oral lichenoid lesions (OLLs) are indistinguishable from oral lichen planus, except that causative factors can be identified, including contact allergy to amalgam. In a Thai study of 53 patients with OLLs, 31 (58.5%) reacted to at least one amalgam component, most commonly to mercury, followed by copper sulfate. Amalgam replacements were performed in 10 patients. Clinical improvement was observed in all cases with complete healing in 3 cases [49]. In a larger series from London (*n* = 115), 76% of cases with an initial erosive or mixed lichenoid reaction and 60% of those presenting with white reticular or plaque-like pattern improved after the replacement of dental restorations with alternative materials; interestingly there was no correlation with contact allergy to dental materials diagnosed in 67.8% of the patients [50]. However, flavouring agents in mouth hygiene products may also be responsible for OLLs [51]. 

### 3.5. Fragrances and Flavours

Several studies in the reporting period addressed the diagnostic performance of fragrance markers used in the baseline series, i.e., FM I and II, hydroxyisohexyl 3-cyclohexene carboxaldehyde (HICC), *Myroxylon pereirae* (balsam of Peru), and, *sensu lato*, colophonium. As one approach, results with these versus the testing of all 26 fragrances presently needing to be labelled in the EU in consecutive patient was compared. In Thailand, 312 consecutive patients were patch tested both with FM I and II and *Myroxylon pereirae* as fragrance markers and all 26 single substances needing labelling in the EU and, interestingly, 15 of overall 84 positive reactions to single fragrance contact allergens were missed by the markers [52]. In the UK, 471 patients had been tested with the aforementioned 3 markers and HICC, and also with 14 essential oils. Eleven of 34 positive reactions to the latter were missed by the markers [53]. Therefore, the limited validity of using the fragrance markers as representation of contact allergy to fragrance contact allergens in general must be considered.

For more than 30 years, FM I has been the most important screening marker for fragrance contact allergy. To examine trends in contact allergy to FM I from 1986 to 2015 in patients with dermatitis, data from 24,168 patients, 7.8% being positive, were analysed. For women, a significant trend was observed in terms of an increase in sensitization to FM I across the three decades. From 2011 to 2015, the prevalence of contact allergy to FM I increased significantly also for men (4.4% vs. 7.3%) compared with the previous 5-year period. Clinical relevance was established in 78.2% of FM I-positive patients with no differences over time [54]. Since the 1990s, FM I contains 5% sorbitan sesquioleate (SSO) as dispersing agent. In principle, contact allergy to this emulsifier which is also used in cosmetics may cause false-positive reactions to FM I; however, according to recent data from Copenhagen, only 0.2% of 4637 consecutively tested patients were positive, translating to 1.4% of all FM I-positive patients [55]. Therefore, the diagnostic value of FM I appears largely unimpaired, but concomitant testing both with FM I and SSO is certainly recommendable. 

FM II, containing 2.5% HICC, is currently recommended to be tested in parallel to HICC 5% pet. in the European baseline series, due to the outstanding importance of HICC sensitisation. To address the necessity of doing so, 2118 patients at five Swedish dermatology departments were consecutively tested with FM II 14% pet., FM II 16.5% pet. (with 5% HICC instead of 2.5%), and duplicate preparations of HICC 5% pet. Altogether, 3.2% reacted to FM II 14%, and 1.5% reacted to HICC. Separate testing with HICC detected 0.3% reactions without concomitant reactivity to FM II, and FM II with 5% HICC did not detect more positive reactions than the standard FM II. According to the authors, separate testing with HICC does not detect a sufficient proportion of patients who react only to HICC to warrant its inclusion in a baseline series [56]. It has, however, been argued that for a follow-up of HICC sensitisation after regulatory action has eventually taken place the continued use of HICC 5% pet. is useful for a certain period.

One topic of current interest in the field of fragrance contact allergy is the patch testing with oxidised fragrance terpenes, namely, hydroperoxides of linalool (Lin-OOHs) and limonene (Lim-OOHs), respectively. In Spain, a multi-centre study of patch testing with Lin-OOHs 1% pet. and Lim-OOHs 0.3%, respectively, in 3639 consecutive patients yielded 4.9% and 5.1% positive reactions [57]. During 2013–2014, 4563 consecutive patients in 12 UK centres were tested with hydroperoxides of limonene 0.3%, 0.2%, and 0.1% pet., and hydroperoxides of linalool 1.0%, 0.5%, and 0.25% pet. The former gave, at 0.3% concentration, positive reactions in 5.3% of patients, irritant reactions in 2.0% and doubtful reactions in 2.4%. Linalool hydroperoxide 1.0% resulted in positive reactions in 7.7%, irritant reactions in 3.9%, and doubtful reactions in 2.9% of the patients. It was recommended that limonene hydroperoxides be tested at 0.3% and linalool hydroperoxides at 1.0% in the British baseline patch test series [58]. In a single-centre US study, 19/96 patients patch tested to Lim-OOHs and Lin-OOHs reacted positively, i.e., a proportion similar to that observed in Europe [59]. Patch testing with oxidized *R*-limonene 3.0% [containing Lim-OOHs 0.33%] and oxidized linalool 6% (Lin-OOHs 1%) in pet. in 2900 consecutive dermatitis patients in Australia, Denmark, Singapore, Spain, Sweden, and the United Kingdom yielded positive reactions in 281 patients. Of these, 25% had concomitant reactions to both compounds, whereas 29% reacted only to oxidized R-limonene and 46% only to oxidized linalool. As the majority of the patients reacted to only one of the oxidation mixtures, the specificity of the reactions is supported [60]. Such specificity is also supported by the results of a database study of consecutively patch tested eczema patients (*n* = 3843) from 2012 to 2015, tested concomitantly with p-phenylenediamine (PPD), Lim-OOHs and Lin-OOHs, where the association between positive reactions was not stronger than expected by chance [61]. In the period 2010 to 2015, the prevalence of sensitization to the 26 fragrances, and concomitant reactivity to FM I and/or FM II was addressed by the same authors; of 6004 patients, 940 (15.7%) were fragrance-sensitized, mostly to Lin-OOH (3.9%), *Evernia furfuracea* (3.0%), Lim-OOH (2.5%), and HICC (2.1%) [62]. As in that study, also the analysis of 4430 patients patch tested on the other side of the Øresund, in Lund/Malmö, pointed to the risk of missing fragrance contact allergy when testing just with the mixes in the baseline series [63] which has been identified in several studies, see also the beginning of this section.

While studies as those reported above seem to point to a considerable contact allergy problem, it has been possible to link exposure to the hydroperoxides with actual ACD only in some well-documented cases. One concerns a 7-year-old Canadian girl, who developed severe eyelid dermatitis after using the PERT PLUS^®^ shampoo of her father, which contained linalool, and low levels (0.2 µg/g) of Lin-OOHs. Avoidance of this shampoo as single intervention led to resolution of her dermatitis [64]. A Swedish study with oxidised lavender oil (6% pet.), i.e., a preparation not only including Lin-OOHs, but also other hydroperoxides, had an even higher yield of positive patch test reactions (2.8%) in 1693 consecutive patients and demonstrated that only 56% of the positive patients concomitantly reacted to the main hydroperoxides, Lin-OOHs and linalyl acetate [65]. 

Essential oils constitute a major source of exposure, according to numerous case reports, but also due to the fact that they are used, in substantial amounts, for the formulation of fragrances for cosmetic products, or fine fragrances. A combined study of North American Contact Dermatitis Group (NACDG) (2009–2014) and the Information Network of Departments of Dermatology (IVDK) (2010–2014) in 13,398 and 48,956 consecutive patients, respectively, identified 1.4% of patients positive to one or more of the natural extracts, but not to one of the fragrance markers of the baseline series. Prevalences were as follows: *Santalum album*, 1.4% IVDK; *Cananga odorata*, 1.1% NACDG, and 2.4% IVDK; *Jasminum* species, 0.7% NACDG, and 1.4% IVDK; *Mentha piperita*, 0.9% NACDG; *Lavandula angustifolia*, 0.3% NACDG; and *Melaleuca alternifolia*, 0.3% NACDG [66]. Of note, essential oils are also found in products labelled “natural”, e.g., insect repellents [67]. 

Sometimes, impurities from the synthesis of a substance are the actual sensitiser, and not the substance itself (which opens possibilities of purification as a means of preventing sensitisation). Concerning the synthetic fragrance Majantol^®^, this is, however, apparently not the case: a study including 53 patients reacting positively out of 8005 consecutive patients tested demonstrated no discordance of test results between a “normal”, unpurified batch including low levels of an organochloride, and a newly developed purified batch of Majantol^®^ [68]. A series of 3 adolescents from the US illustrates that Majantol^®^ is a relevant contact allergen, which is difficult to suspect—and later to avoid—as it is currently not covered by the mandatory labelling in the EU, and certainly not elsewhere [69]. 

In 3 paediatric patients with atopic eczema, including the scalp and leading to circumscribed alopecia, therapeutic exposure to balm-containing remedies (including a fluocinolone ointment) had apparently induced contact allergy to *Myroxylon pereirae* (balsam of Peru), and avoidance of these products led to resolution of scalp inflammation and alopecia [70]. The repeated application of “insect repellent wipes” in a 3-year-old Italian boy had induced widespread dermatitis. Patch testing was positive to the wipe “as is” and also to hydroxycitronellal; liquid chromatography-mass spectrometry analysis of the wipes could, unfortunately, not distinguish between the presence of hydroxycitronellal and the declared ingredient citriodiol [71]. Passengers (and drivers) in Uber cars (or in other public spaces, for that matter) may or may not enjoy scents transmitted via aerosols. In 1 driver and 4 passengers, airborne ACD to such scents was diagnosed in Miami, FL [72]. 

The presence of fragrances as constituent not only of cosmetics, but also pharmaceutical topical products has been examined by a Brazilian market survey, finding almost 30% of all 305 such products to contain fragrances [73]. 

Since 2005, 26 fragrance contact allergens have been mandatory to label in cosmetic products within the EU if present at 10 ppm or above in leave-on and 100 ppm or above in wash-off cosmetics. To examine exposure, based on ingredient labelling, a novel, non-profit smartphone application (app), developed to provide information to consumers about ingredients of cosmetic products, was used. The largest product categories investigated were “cream, lotion and oil” (*n* = 1192), “shampoo and conditioner” (*n* = 968) and “deodorants” (*n* = 632). As in previous investigations assessing labelling information, linalool (49.5%) and limonene (48.5%) were found most often among all investigated products. HICC was found in 13.5% of deodorants. Six of the 26 fragrance substances were labelled on less than one per cent of all products, including the natural extracts *Evernia furfuracea* (tree moss) and *Evernia prunastri* (oak moss). It was thus confirmed that consumers are often widely exposed to multiple, well-established fragrance contact allergens through various cosmetic products intended for daily use [74]. 

If a weak (fragrance) contact allergen is found in many products, the patient may need to sort out many of his cosmetic products, as illustrated by a 60-year-old Spanish patient, who suffered from periorbital eczema due to sensitisation to benzyl salicylate, and identified 5 of her own products needing to be discarded [75]. *L*-Carvone is a known contact allergen and has been found (as constituent of mint flavour) in 64 of 66 Swedish brand toothpastes examined; in 10 of these the concentration exceeded 0.1% [76]. In case of contact allergy to such a widely used agent, formulation of a toothpaste by a pharmacy may be considered [77]. It should be noted that mucosal problems may occur (also to other ingredients of toothpastes etc., such as *Salvadora persica* extract [78]), but cheilitis and perioral dermatitis are probably a greater problem caused by toothpaste contact allergens, as also noted in some case reports featured in cosmetic contact allergens.

A novel analysis of the association of pairs of positive-reacting contact allergens with polysensitisation (being allergic to 3 or more contact allergens from the baseline series) has shown that, besides nickel and cobalt allergy combined, coupled reactivity to the various fragrances included in the baseline series is the major driving force for multiple sensitisation, thereby stressing the importance of this group of contact allergens [79]. 

### 3.6. Preservatives

Biocides, e.g., those used as preservatives in cosmetic, household, or industrial products, are invariably also sensitizers; these characteristics seem to be related to the biological activity necessary for their antimicrobial effectiveness. The broad range of uses vs. the quite limited number of biocides which are available, e.g., registered as preservatives in the EU Cosmetics Regulation (EC 1223/2009, Annex V), creates the problem of the same contact allergen being found in diverse occupational and non-occupational products, thus causing difficulties in avoidance of these once sensitized. A survey of 4737 products documented in the Contact Allergen Management Program in the USA demonstrated 23.9% containing 2-phenoxyethanol, followed by 20.8% containing parabens, while MI was found in 12.9% of products—this observation, combined with the frequency of contact allergies, points to the exceedingly low sensitisation risk associated with parabens and 2-phenoxyethanol [80]. A Danish survey found extensive use of isothiazolinones, but also of formaldehyde in various product types used occupationally, which can be regarded as problematic, in particular for the sensitised worker [81]. 

#### 3.6.1. Isothiazolinones

The unprecedented epidemic of contact allergy to methylisothiazolinone (MI) seemed to start to have declined during the reporting period, as reported from Genova, Italy [82], Leeds, UK [83], Lithuania [84] and in a multi-centre study covering 8 European countries, finding an overall prevalence of 6% positive reactions [85]. The relative importance of exposure to rinse-off cosmetics [85,86] and household products is high [82]. The analysis of household products on the Swiss market found that 42.9% of detergents as compared to an average of 7.9% of all cosmetics contained isothiazolinones [87]. Expectedly, other parts of the world where use of MCI and MI is more restricted did not suffer from the above-mentioned epidemic: contact allergy prevalence to MCI/MI remained stable at a low level of 1.8% in consecutive patients in Singapore, which has been attributed to the fact that mainly Japanese cosmetics are popular in Singapore, and these must not contain MCI/MI [88]. Conversely, in Thailand where such habits apparently do not exist, MCI/MI has become the most common preservative sensitiser, with 13.6% allergic in a series of 206 patients tested 2012–2015 [89]. 

As much of the population has been sensitised, (i) exposures beyond the initially causative cosmetics are becoming more relevant and (ii) cross-reactivity to other isothiazolinone preservatives is an important issue. Addressing the latter aspect, it has been found that allergic reactions to octylisothiazolinone (OIT) were strongly associated with extreme reactions to MI, which suggests cross-sensitization, likely dependent on the sensitizing dose. In contrast, reactions to benzisothiazolinone (BIT) were mostly independent [90]. Patch testing with BIT is done with 0.05% and 0.1% mostly in pet., sometimes in aq., and the latter concentration has been suggested to be more sensitive [91]. While at least in Europe neither OIT nor BIT are permitted to be used in cosmetics, other uses and subsequent sensitisation have been reported, e.g., in 2 Belgian printers allergic to BIT in printing ink, and one also in liquid soap [92]. 

The many sources of exposure to MI are partly unexpected: a “100% natural” Konjac^®^ cosmetic sponge contained ~400 ppm MI and caused hand and face dermatitis in an exposed female [93] and temple ACD due to the presence in a designer spectacle frame [94]. In the sponge of a vacuum pump used to treat an ulcer in a patient sensitised to MI by previous accidental exposure, 754 ppm MCI and 315 ppm MI were found [95]. Undisclosed presence was noted in adhesive labels, leading to ACD of the exposed fingers [96], partially airborne ACD after using ironing water preserved with MI in 4 British patients [97], soothing and cooling gel pads applied to the lower lids during application of lash extensions [98], airborne ACD accompanied by a dry cough in a construction worker [99], (partly airborne) ACD due to a stainless steel cleaning spray [100], foot dermatitis due to a shoe glue preserved with MI [101], occupational ACD in two gynaecologists due to MI in ultrasound gel [102], chronic dermatitis of the trunk and proximal extremities in a 7-year-old girl due to liquid laundry detergent [103], although it has been claimed, based on a small study, that MI cannot be detected in the laundered fabric [104]. Newly painted rooms, with airborne exposure to MI for several weeks, is a well-known cause of elicitation of ACD by MI [85,105,106,107], partly leading to very severe and atypical clinical presentations [108]. The need of a complete declaration of MI (and all isothiazolinones) has been illustrated by the case of a 9-year-old boy with widespread airborne ACD where a (online accessible) product data sheet informed about the presence of MI, MCI, and BIT and thus the cause was found [109]. 

Use in leather is addressed in the section “textile and leather contact allergens”.

#### 3.6.2. Other Preservatives

Formaldehyde has largely been replaced as a preservative in cosmetics, however, certain uses and exposures can still be identified, e.g., by a study covering 1996 to 2012 and 23,774 patients in North-east Italy, where woodworkers, professional drivers, textile workers, and healthcare workers have been identified to have an increases risk of sensitisation, compared to “white collar workers” [110]. A range of new biocide (preservative) contact allergens has been described in the last 2 years, see Table 1.

While formaldehyde itself is not used much as preservative, so-called formaldehyde-releasers are widely used. A Swedish study examined cosmetics from 10 formaldehyde-allergic and 30 non-allergic patients as controls with the chromotropic acid spot test, which is a semi-quantitative method measuring the release of formaldehyde. Formaldehyde was found in 58 of 245 (23.7%) products, including 26 leave-on products, 17 of which did not declare containing formaldehyde or formaldehyde releasers. Similar observations were made in rinse-off products. Therefore, it was recommended that cosmetic products used by formaldehyde-allergic patients that are not declared to contain formaldehyde or formaldehyde-releasing preservatives should be analysed [111]. In a further study from this group, 15 formaldehyde-allergic individuals and a control group of 12 individuals without contact allergy to formaldehyde and formaldehyde releasers performed a repeated open application test (ROAT) over 4 weeks on pre-irritated skin areas with four different moisturisers releasing formaldehyde in concentrations that had been determined as >40, 20–40, 2.5–10, and 0 ppm by the chromotropic acid (CA) spot test. Dimethylol dimethyl hydantoin (DMDM hydantoin) was used as a formaldehyde releaser in the moisturisers. Nine of the 15 formaldehyde-allergic individuals showed a reappearance or worsening of dermatitis on the formaldehyde exposed areas, with all control tests being negative. Therefore, the low concentrations of formaldehyde often found in skincare products by the CA method are sufficient to worsen an existing dermatitis in formaldehyde-allergic individuals [112]. 

Moisturisers under the trade name Apobase^®^ marketed in Finland underwent a formulation change in terms of introduction of Phenostat™, a mixture of phenoxyethanol, caprylhydroxamic acid, and methylpropanediol. Thirty-nine patients with suspected contact allergy to Apobase^®^ creams or lotion were patch tested with old and new Apobase^®^ formulas and their preservatives, and positive reactions were found only to the new Apobase^®^ formulas, Phenostat™, and caprylhydroxamic acid or its potassium salt [113]. The importance of this new contact allergen, caprylhydroxyamic acid, needs further investigation in countries where this substance is found on the market.

Methyldibromo glutaronitrile (MDBGN) has been prohibited in cosmetics in the EU, which has led to a decline of incident sensitisation, as witnessed in results from Northern Italy, according to which, sensitisation prevalence peaked between 1999 and 2007, and declined thereafter [114]. However, cosmetics bought outside the EU may still contain MDBGN, and non-cosmetic products in the EU, as illustrated by 2 patients from Copenhagen, one sensitised to a leather detergent, the other to a sunscreen bought in Guatemala [115]. 

### 3.7. Plastic Contact Allergens

Currently, an increase of contact allergy to (meth)acrylates is observed in many countries, which is attributed to the increasing use of various types of artificial nails, e.g., from Leeds, UK [116], or St. John’s, London [117], or in 230 patients in a multi-centre study involving 13 Portuguese departments [118]. In this latter study, comprising 55 nail technicians, 56 consumers, and 119 with mixed exposure, mostly chronic hand eczema (93%) was observed. The most common sensitizers were: 2-hydroxyethyl methacrylate (HEMA), which was positive in 90% of the tested patients, 2-hydroxypropyl methacrylate (HPMA), which was positive in 64.1%, and ethyleneglycol dimethacrylate, which was positive in 54.5%. During a similar period, 2353 patients were patch tested in a Spanish multi-centre study, and 43 (1.82%) were diagnosed with ACD caused by (meth)acrylates in long-lasting nail polish; all were female, and all had hand dermatitis [119]. A Polish study found contact dermatitis reported by 43% of nail technicians participating in a survey [120]. 

A series of 8 patients was diagnosed with contact allergy to several (meth)acrylates in a particular home-use UV-curing polish in 5 Swedish departments [121]. A shift from industrial (or medical and dental [122]) exposure to cosmetic exposure has been clearly noted in a 2002–2015 series from Birmingham, UK, in which 52 patients of 475 tested positive [123]. Of note, the parent acids, namely, acrylic and methacrylic acid, do not appear to be important contact allergens according to some authors—as witnessed by altogether negative results in 768 consecutive Swedish patients [124]—but only considering their esters.

Epoxy resin systems (ERSs), consisting of resins, reactive diluents, and hardeners, are indispensable in many fields of industry. A retrospective analysis of data from the Information Network of Departments of Dermatology (IVDK), 2002–2011, demonstrated that almost half of the patients sensitized to epoxy resin were additionally sensitized to reactive diluents or hardeners. Among the reactive diluents, 1,6-hexanediol diglycidyl ether was the most frequent contact allergen, followed by 1,4-butanediol diglycidyl ether (both often cross-react [125]), phenyl glycidyl ether, and *p-tert*-butylphenyl glycidyl ether. Among the hardeners, m-xylylene diamine (MXDA) and isophorone diamine (IPDA) were the most frequent contact allergens. According to the calculated exposure-related frequency of sensitization, MXDA seems to be a far more important sensitizer than IPDA. As up to 60% of the patients sensitized to hardeners and 15–20% of those sensitized to reactive diluents do not react to epoxy resin, a complete epoxy resin series must be patch tested from the start [126]. New ESR components are evolving and need to be patch tested individually, where indicated, such as in a case of ACD to 2-methylpentane-1,5-diamine [127], or occupational ACD in a spray painter caused by hydrogenated formaldehyde–benzenamine polymer (FBAP) in epoxy hardeners [128]. A new application for ESR resulting in occupational ACD has been illustrated by 2 Belgian workers in the 3-dimensional printing industry [129]. 

A novel group of contact allergens: *N*-(2-phenylethyl) derivatives of the reactive amine 1,3-benzenedimethanamine (1,3-BDMA), were described in 6 patients with occupational contact allergy to these, 4 being spray painters who used epoxy paints, 1 a floor layer who handled a variety of epoxy coatings, and 1 a worker in epoxy hardener manufacture. Because of the lack of a commercially available patch test substance, this has to be prepared in-house, best as a dilution series, in this case starting well below 1%, down to 0.0001% [130].

ACD caused by polyester resin is comparatively rare. Between 1994 and 2015, 11 patients were diagnosed at the Finnish Institute of Occupational Health (FIOH), 5 car painters and repairers, the remainder working in some industrial occupations with contact to putties or paints. As commercial patch test substances of polyester resin putties are lacking, patients’ own products need to be tested to diagnose sensitisation. Conversely, contact allergy to cobalt salts used in several types of polyester resin product as accelerators [131] can be diagnosed with the baseline series. Contact allergy to diisocyanates, which are notoriously difficult to test as they rapidly degrade, can quite reliably be diagnosed by patch testing with the corresponding (stable) amine. In Northeast Italy, from 1996 to 2012, 24,056 consecutive patients with suspected ACD were patch tested in eight departments of dermatology and occupational medicine. Of these, 2.5% were sensitised to diaminodiphenylmethane with a decreasing trend. Mechanics and chemical industry workers had a significant higher risk of being sensitized [132]. 

Among formaldehyde-based resins, *p*-*tert*-butylphenol formaldehyde resin is included in the baseline series, and is still important as a widely used adhesive, e.g., in orthopaedic braces [133]. Other derivatives are tested in special series, guided by exposure analysis. In the FIOH, the resol-type phenol formaldehyde resin (PFR 2) has been added to the baseline series of this specialised institution, and in 2012 to 2016, yielded 1.6% positive reactions, 70% of which were clinically relevant, and therefore the contact allergen is evidently important. It was therefore recommended to evaluate its importance in general dermatology clinics [134]. 

Plastic (and other) contact allergens in medical devices are covered in the respective section below.

### 3.8. Rubber Contact Allergens

The baseline series includes a number of single contact allergens or mixes to screen for rubber contact allergens: thiuram mix (tetramethylthiuram monosulfide, tetramethylthiuram disulfide, tetraethylthiuram disulfide and dipentamethylenethiuram disulfide), mercapto mix (morpholinyl mercaptobenzothiazole, dibenzothiazyl disulfide, *N*-cyclohexyl-2-benzothiazyl-sulfenamide), 2-mercaptobenzothiazole, and *N*-isopropyl-*N′*-phenyl-*p*-phenylenediamine (IPPD). However, despite this relatively broad screening in different classes of additives, additional testing of a dedicated rubber series not only containing the break-down of above mixes, but also further substances, does yield a large share of additional positive reactions and is thus recommended in patients with suspected (occupational) rubber allergy to avoid a diagnostic gap [135]. 

Among the rubber vulcanization accelerators, thiurams and dithiocarbamates are structurally related and important sensitizers, e.g., in healthcare workers [136]. In 155 patients who reacted positively to at least one thiuram or dithiocarbamate in the rubber chemical series in the FIOH, 34 reacted positively to some dithiocarbamate derivatives, and this was always related to thiuram allergy, i.e., from these results, screening with carba mix would appear unnecessary [137], although it has been recommended, based on a relatively high share of (partly relevant) positive reactions not diagnosed otherwise [138] and which has been increasing in the recent years [139]. Even short contacts as with the rubber glove of a treating dentist can cause ACD in the patients, as illustrated by 2 Danish cases [140]. Moreover, airborne exposure has apparently caused face dermatitis in a 56-year-old lab worker sensitised to thiuram mix and tetraethylthiuram disulfide in particular [141]. Such types of exposure, beyond the evident direct contact with the glove-donned hands, may have contributed to an observed association between rubber allergy and facial dermatitis [142]. 

Several derivatives of *p*-phenylenediamine are used as colouring antioxidants in rubber; the marker of this class in the baseline series is IPPD or a “black rubber mix” (containing IPPD, *N*-cyclohexyl-*N′*-phenyl *p*-phenylenediamine and *N*,*N*′-diphenyl *p*-phenylenediamine). In a Danish otorhinolaryngologist the ocular pieces of a newly bought otomicroscope caused periorbital ACD by these antioxidants and led the physician to revert to the formerly used model [143]. An Italian motorcyclist suffered from palmar dermatitis due to contact allergy to IPPD contained in his new, heated motorcycle grips [144]. 

Thiourea derivatives are less common sensitisers in rubber products, including the chloroprene (neoprene™) type of foamy rubber. A Danish fisherman suffered from severe bullous dermatitis of his feet after wearing new rubber boots made of a polymer blend of neoprene with natural rubber latex. Later patch testing revealed sensitisation to diethylthiourea (1% pet., +++ reaction) as the cause and questioning revealed a putative episode of ACD under after wearing a knee brace which probably had been the sensitising event [145]. Diethylthiourea can decompose to the sensitizer ethyl isothiocyanate. Liquid chromatography/mass spectrometry and solid-phase microextraction/gas chromatography were used for determination of organic thioureas and isothiocyanates in rubber chloroprene rubber samples. All 8 patients allergic to diphenylthiourea reacted to phenyl isothiocyanate, 2/8 to phenyl isocyanate. Four patients allergic to diethylthiourea reacted at retest; diethylthiourea was detected in all chloroprene rubber samples, with levels of 2–1200 nmol/cm^2^. At 35 °C, ethyl isothiocyanate was emitted from all samples. Patch-test preparations of diethylthiourea, diphenylthiourea, and dibutylthiourea all emitted the corresponding isothiocyanate, with diethylthiourea showing the highest rate of isothiocyanate emission. In conclusion, thiourea compounds are degraded to isothiocyanates, which are generally strong or extreme sensitizers, thus acting as prehaptens, or in the case of diphenylthiourea, as a prohapten. This process occurs in both chloroprene rubber products and patch-test preparations [146]. 

While additives not essential to the production or shelf-life of rubber products are not as specific, they may represent unusual or hidden sources of exposure or sensitisation, as illustrated by the case of a renal transplant nurse who developed hand dermatitis after blue nitrile gloves had been introduced at the workplace. Elaborate testing and analysis with thin-layer chromatography (TLC) finally revealed contact allergy to Pigment Blue 15 as cause of the problem; indeed reverting to the use of white gloves cleared the problem [147].

A new contact allergen in shin pads that was responsible for severe contact dermatitis in a young football player was identified by employing high-performance liquid chromatography (HPLC) of samples of shin pads. Patch tests with pieces of shin pads and with acetophenone azine, a chemical substance identified by HPLC in the foam of the shin pads at ~20 µg/g, was strong positive down to 0.001% acetophenone azine in acetone. Interestingly, the substance is not added during the production of ethyl vinyl acetate (EVA) foam, but is apparently newly formed [148]. In two additional cases, also shin pads in one case, and sneakers in the other, were the cause of a partly spreading severe ACD [149]; in another Belgian case, again shin pads and later sports shoes had caused severe ACD in a 29-year-old hockey player, and again, acetophenone azine was identified as the contact allergen [150]. 

### 3.9. Pharmaceutical Contact Allergen

Generally speaking, contact allergens found in pharmaceutical products applied topically contribute a large number of contact allergies in the spectrum of patch test diagnoses. A tertiary referral centre in Leuven, Belgium, for instance, found that between 1990 and 2014, 17.4% of all 14,911 patients consecutively tested were allergic to one or several of such contact allergens, albeit with a slightly declining trend over time [151]. Novel contact allergens reported in the recent years are listed in Table 2.

Corticosteroids are a challenging class of contact allergens—their intrinsic pharmacodynamic activity possibly partly impeding or modifying elicitation (including patch test) reactions, ideal vehicle and test concentration being debated, and often causing positive patch test reactions only at around D7, which is not included in all reading schemes. The NACDG had tested 17,978 patients between 2007 and 2014 with 6 corticosteroids, of which tixocortol-21 pivalate caused 2.3% positive reactions, budesonide 0.87%, hydrocortisone-17 butyrate 0.43%, clobetasol-17 propionate 0.32%. and desoximethasone 0.16% [152]. 

In 16 ophthalmological patients undergoing repeated intravitreal injections with anti-VEGF contact dermatitis had developed in the Ghent department; 9 patients reacted to phenylephrine, 5 to iso-Betadine^®^ ophthalmic solution and 3 patients to sodium metabisulfite [153]. Betadine^®^ is a brand name for povidone iodine-containing solutions. Patch testing is usually performed with PVP iodine 10% aq.; however, false-positive and irritant reactions are quite common. Nevertheless, lowering the patch test concentration proved to impair diagnostic sensitivity in a study of 79 patients tested with different dilutions, therefore, either a ROAT or additional patch test with iodine 0.5% pet. is recommended [154]. 

Perianal dermatitis and pruritus ani should be investigated by patch testing, including products such as anti-haemorrhoidal creams used, and incriminated, by the patient, as stressed by the results of a series of 150 patients obtained at St. John’s Institute of Dermatology, London. Relevant contact allergens included MI found in wet wipes, local antibiotics, anaesthetics, and fragrances [155]. Another case with sensitisation to topically applied clotrimazole, leading to anogenital dermatitis and subsequent maculopapular rash after systemic administration of fluconazole illustrates the potential for systemic elicitation after topical induction to a cross-reacting contact allergen [156]; the perianal region, the axilla, is particularly prone to contact allergen penetration due to the local factors of occlusion and maceration.

Topically applied amide type local anaesthetics, such as dibucaine (cinchocaine) or ester type drugs such as benzocaine, are another well-known source of sensitisation related to anogenital application. However, lidocaine is also used as an injectable drug, and the question whether contact allergy (as demonstrated by a positive patch test) will lead to systemic reaction has been addressed by a US study in 756 patients, of whom 13 were positive to lidocaine 15% pet.; 3 showed delayed positive reactions to subcutaneous challenge, but, according to the authors, systemic use is probably safe for those with neither a delayed nor an immediate reaction after provocation testing [157]. 

A 40-year-old nurse working in a treatment centre for addicts developed acute, relapsing, presumably airborne face dermatitis when handling heroin (diacetylmorphine) and morphine; both opiates were strongly positive at patch testing, which was negative to oxycodone, methadone, and codeine, illustrating a very specific sensitisation pattern [158]. 

Two employees of a chemical plant producing, among other substances, ethylene diamine outdoors, became sensitised to this substance, but also to related amines such as diethylene diamine and triethylene tetramine, which necessitated a change of job [159]. 

### 3.10. Cosmetic Contact Allergens

ACD due to contact allergens contained in cosmetics often affect the face [160], either because they are directly applied to the face, or spread or are transferred to the face—one example being hair dyes. Oxidative hair dyes are a well-recognised source of exposure to potent contact allergens, namely, their basic substances and coupling agents. In view of the fact that many consumers, including those sensitised, prefer to nevertheless dye their hair (even if expecting symptoms of ACD after doing so [161]), alternative preventive strategies are being developed. One approach is to protect the surrounding glabrous skin by the application of a topical antioxidant (ascorbic acid) prior to hair dyeing, as has been quite successfully tried in 2 proof-of-principle studies [162,163]. The other approach is the development of presumably less-sensitising derivatives of e.g., *p*-phenylenediamine (PPD). Although salts such as PPD-dihydrochloride are sometimes also used, patch testing with the free PPD base is currently preferred, as PPD-dihydrochloride at 1% pet. is less sensitive than PPD 1% pet. [164]. Using this contact allergen preparation, the ESSCA network tested 99,926 patients in the period 2002–2012 and found no time trend, but significant differences between countries, with the highest sensitisation prevalence observed in Lithuania, and the lowest in Slovenia. Moreover, strong or extreme positive reactions were noted more often in the south of Europe than in the centre and north, indicative of a higher exposure, e.g., by (more frequent use of) darker shades [165]. Results from Budapest, Hungary, obtained in 3631 consecutive patients tested between 2007 and 2014 revealed (i) a higher prevalence than otherwise reported from central Europe, namely 5.8%, and (ii) an increasing share of younger patients, probably related to changing fashion trends [166]. In contrast, for instance, a retrospective study from The Netherlands covering 1994–2014 found a PPD contact allergy prevalence of 2.9% [167]. On the population level, the average sensitisation prevalence across 5 European countries was 0.8%, according to results from the EDEN study [168]. 

In Japan, ACD caused by hair colouring agents is a considerable problem for hairdressers and also for consumers; ~7% positive patch test reactions to PPD have been diagnosed in consecutive patients in the recent years. Investigating a patch test series of 19 hair cosmetic contact allergens, including PPD, Bandrowski’s base, cysteamine HCl, and ammonium thioglycolate, 203 patients were tested in 14 Japanese hospitals. Positive reactions were most frequently seen to PPD (35.1%) but *p*-methylaminophenol and *o*-aminophenol were also often positive, both in the PPD-positive and in the PPD-negative patients. Based on this study, 13 contact allergens were recommended for a Japanese hairdresser series [169]. A double case report from Dundee, UK, points to eyelash and eyebrow tints as source of PPD-related severe ACD [170]. According to a Korean study with 31 patients with positive reactions to PPD and/or toluene-2,5-diamine (PTD), these may safely use hair dyes based on gallic acid and ferrous sulfate, notwithstanding some irritation by these in some patients in a use test [171]. 

Preservatives contained in 1000 different cosmetics sold in Thai markets were analysed concerning ingredient labelling. International brand cosmetics were more likely to contain preservatives other than formaldehyde-releasing ones than domestically produced brands [172]. Similar differences were observed with hair dyes, where domestic production apparently included a lesser proportion of potent skin sensitisers than international brands [173]. This observation points to the global differences of product formulation which may be more important in less-regulated markets. Interestingly, a notable proportion of “natural” hair dyes does contain well-known contact allergens such as *m*-aminophenol or Acid Violet 43, according to a Swedish survey of 92 such products [174]. Wet wipes are an important source of sensitisation, owing to the specific anatomical conditions and to the fact that these are in fact “leave-on” cosmetics. Between 2011 and 2014, 79 patients were identified by NACDG members in whom wet wipe-related contact allergens were identified, mostly MI (59%), MCI/MI (35.6%), 2-bromo-2-nitropropane-1,3-diol (bronopol; 27.4%) and iodopropynyl butylcarbamate (12.3%) [175]. 

The prevalence of contact allergy to some emulsifiers commonly found in topical products was assessed in 310 Italian patients, of whom 50 (16%) had positive patch test reactions to at least one of 15 emulsifiers tested. Lauryl polyethylene glycol/polypropylene glycol-18/18 methicone gave 26 positive reactions, glyceryl oleate 19, myristyl alcohol, and Amerchol™ L101 11. While contact allergy to emulsifiers may indeed be more frequent than reported [176], the possibility of irritant/false-positive reactions to these needs to be considered. In a US study involving 47 patients previously testing positive to various surfactants 3 novel surfactants were patch tested, of which isostearamidopropyl morpholine lactate (0.5 and 1% aq.) yielded 11 positive (+ and ++) and 21 doubtful reactions. Hence, this substance was identified as a putatively important contact allergen needing further investigation [177]. Of note, screening to alkyl glucoside surfactants is not possible by using just one of the derivatives, as demonstrated by a study with 48 patients reacting either to decyl or lauryl glucoside (with 41% definite or probable relevance), but only 65% of the patients reacted to both derivatives [178]. 

The finding of a significant association between contact allergy to preservatives (formaldehyde, as marker for formaldehyde-releasers, MCI/MI and MI) and fragrances (FM I and II) in a Swedish study in 2165 patients [179] possibly points to cosmetics as source of exposure to both groups of contact allergens.

For two reasons, use of alcoholic hand rubs is preferred over use of liquid soaps (mostly relevant in an occupational context): irritation by alcohol is less than by detergents, and liquid soaps contain various constituents such as preservatives, emulsifiers, and fragrances which may cause contact allergy, as illustrated by experience from an Australian occupational dermatology referral centre [180]. 

Octocrylene is a commonly used UV-filter and a relatively frequent cause of photo contact allergy, particularly in those sensitised to ketoprofen [181]. However, according to patch test results (not photo patch test) in 2577 consecutively tested patients in the IVDK network, “normal” sensitisation is rare, observed in just 2 patients (0.08%) [182]. Salicylates form a heterogeneous group of substances, partly used as fragrance (benzyl salicylate), as a UV-filter in sunscreens (octyl salicylate and homosalate), or as other cosmetic ingredients (menthyl and phenyl salicylate). Results from one US department point to possible cross-reactivity (except with homosalate), but also to a need of further investigating this hitherto neglected topic [183]. 

In the wide arena of cosmetics, tattoos have a special role as a permanent perceived enhancement of appearance. Tattoo inks are quite ill-defined, beyond the use of carbon black for the standard black colour. Besides actual dyes, excipients may play a role as contact allergens, as shown by a 26-year-old patient with a large black tattoo, in which pruritic papules had appeared a few weeks after tattooing. Both the original ink and shellac (20% eth.) used as binding agent in such inks elicited strong positive reactions [184]. Temporary tattoos, usually based on henna, have gained popularity e.g., as a holiday souvenir for adolescents—alas, often with the life-long side-effect of sensitisation to PPD, as the “henna tattoos” are often adulterated with PPD (or resorcinol [185]) to increase darkness. A new temporary tattoo made from the Jagua fruit (*Genipa americana*), yielding a dark-blue colour lasting for a few weeks is being marketed as PPD-free alternative. However, a first case report has described putative ACD to a Jagua tattoo [186], and a second case with thorough chemical analytical work-up and patch testing with pure substances identified genipin, patch test positive at 0.5, 1, and 2% in dimethylsulfoxide/water 1:1 *v*/*v*, as the culprit contact allergen [187]. A considerable number of other cosmetic contact allergens has been described in the period of analysis; findings are summarized in Table 3.

### 3.11. Woods and Plants

A 51-year-old Chinese patient working in the processing of *Gingko biloba* fruits wore rubber gloves to protect her hands but developed work-related ACD on her arms and in the face. A positive ++ reaction with an extract of sarcotesta of *G. biloba* confirmed the diagnosis, which apparently is not uncommon where *G. biloba* fruits are harvested [188]. An Italian hunter developed severe dermatitis of his face, recurring after autumn hunting trips in Apulia. Patch testing demonstrated contact allergy to *Dittrichia viscosa* (L.) Greuter (sticky fleabane) tested as “wetted and pounded” leaves, negative in 20 healthy volunteers [189]. The fact that western Christmas habits may be a problem for those sensitised to colophonium was illustrated by a Danish 28-year-old atopic woman, who noted a severe bout of facial dermatitis after decorating a Christmas tree. Patch testing was not only (strongly) positive to colophonium, but also to spruce needles tested “as is” [190]. 

A woodwork teacher was able to link four episodes of face dermatitis to occupational exposure to sawdust of *Dalbergia retusa*. Dust and scrapings from the wood material applied to the skin “as is” yielded a ++ patch test reaction at D2 and D3 [191]. In a 62-year-old Spanish parquet fitter, airborne exposure to dust of *Apuleia leiocarpa* had led to face and chest dermatitis, sensitisation verified by patch testing [192]. Collectively, these results point to the well-known sensitization potential of, in fact, several tropical hardwoods. However, also softwoods can cause contact allergy, as illustrated by 3 Finnish cases who became sensitised to Western red cedar (*Thuja plicata*) in a sauna. Interestingly, colophonium included in the baseline series was positive in just one of the cases, while sawdust moistened with water caused ++ reactions and provided the diagnosis [193]. 

Decorative plants, flowering or not, may occasionally cause contact allergy. While this is generally quite rare, occupational exposure may be much more intense. This is illustrated by a 49-year-old Danish skilled gardener cultivating Boston fern (*Neprolepsis exaltata* “Bostoniensis”) and developing ACD limited to the exposed 2 fingers and dorsum of the right hand; a patch test with fern pinna was positive—and negative in 31 controls [194]; it is mandatory to rule out irritancy by patch testing controls with such materials where little evidence concerning optimum patch test application exists. In some plant materials, and also other contact allergens for that matter, the potent allergens contained may cause active sensitization; therefore, caution when patch testing and prior consultation of relevant reference works or experts is warranted. The identification of the contact allergen(s) in plants is a challenging task, combining analytical chemistry and diligent patch testing of patients found sensitised to the whole extract, or plant part(s). Concerning the cushion bush (*Leucophyta* brownii Cass.), 6 of 7 sesquiterpene lactones isolated yielded a varying number of positive reactions in 11 patients sensitised to the bush, thus further characterising the chemical contact allergen profile of this native Australian bush [195]. In a French case, hand and neck dermatitis in a young female first appeared after buying a rabbit as a pet, but was eventually attributable not to animal, but plant contact allergens: patch testing revealed contact allergy to sesquiterpene lactone mix in the baseline series, and to chicory (*Cichorium intybus* var. *foliosum*), tested “as is”, i.e., to the rabbit feed used [196]. 

Another possibility of exposure to plant contact allergens is ingestion—as folk remedy or alternative medicine, or just for pleasure. A 26-year-old atopic female presented with a widespread and itchy maculopapular rash, which, after careful history taking and extensive patch testing, could be explained by the ingestion of a herbal tea containing *Cinnamomum zeylanicum*, and by strong positive reactions to cinnamal and cinnamyl alcohol [197]. 

### 3.12. Textile and Leather Contact Allergens

A 35-year-old male textile worker wearing dreadlocks and applying a black dye to wool developed a recurrent dermatitis of exposed skin areas and the neck, which was finally found to be ACD to several reactive dyes. Black powder had collected in his hair and contributed to on-going neck dermatitis, as became evident when cutting his hair—which finally led to resolution of dermatitis [198]. Textiles ACD often resembles atopic dermatitis, which makes diagnosis difficult, as illustrated by a 39-year-old Danish male with a history of atopic dermatitis, whose superimposed textile dermatitis due to sensitisation to various PPD-related dyes had been diagnosed only because additional work-related hand dermatitis was suspected, and a patch test was initiated [199]. 

Freshly tanned leather is often transported over long distances and, if moist, has to be preserved. This is the putative explanation for observing contact allergy to biocides induced by various leather products, e.g., octylisothiazolinone in a series of 6 Belgian patients [200] or 2 elderly French patients with widespread ACD at the contact sites with their leather sofas, due to OIT sensitisation [201]. In a 42-year-old Japanese woman, a new pair of black trousers caused ACD of the legs, which was attributable to 4,5-dichloro-2-n-octyl-4-isothiazolin-3-one (DCOIT) found in these; contact allergy was diagnosed by a + reaction to DCOIT 0.05% pet. and ++ to DCOIT 0.1% pet. [202]. Occasionally, dyes used in the manufacture of shoes may induce contact allergy, as in the case of a 40-year-old man with severe chronic dermatitis of the dorsa of his feet, which was attributed to multiple sensitisation to reactive dyes (including Reactive Black 5, Blue 21, Blue 238, Orange 107, Red 228 Violet 5) contained in 2 pairs of textile shoes used—avoidance of these led to complete resolution within 8 weeks [203]. 

A retrospective analysis of IVDK data of the years 2007–2014 identified 3207 patients tested with a textile dye series. The highest prevalence of positive reactions was observed for *p*-aminoazobenzene (5.1%) and PPD (4.5%), followed by Disperse Orange 3 (3.1%), Disperse Blue 124 (2.3%), Disperse Blue 106 (2.0%), Disperse Red 17 (1.1%), and Disperse Yellow 3 (1.1%), partly with concomitant reactions. Patch testing with the patients’ own textiles was performed in 315 patients, with positive reactions in 18 patients, mostly due to tight blue or black textiles with immediate skin contact—only 2 of these patients also reacted to textile dyes from the test series. Hence, patch testing with PPD, a textile dye series and patients’ own clothing is necessary for diagnosis [204]; moreover, the European baseline series includes a textile dye mix for some years. This latter mix yields a considerable proportion of positive patch test reactions in consecutive patients, such as 2.8% and 3.1% in Vilnius, Lithuania and Malmö, Sweden, respectively [205]. 

### 3.13. Medical Devices

Glucose sensors and insulin pumps provide an unprecedented accuracy of glycaemic control. However, several case reports and case series point to adverse reactions to different plastic resin contact allergens in these systems, which stay on the skin for prolonged durations and thereby may facilitate sensitisation. These include ACD due to cyanoacrylates (ethyl cyanoacrylate) in a 2-year-old [206], a 9-year-old [207] and a 15-year old [208] diabetic child and 2 adult diabetics [209] which have elsewhere been identified as causes of ACD to surgical glues (e.g., Dermabond^®^ containing 2-octyl cyanoacrylate; [210]). In these cases, a scratch test prior to exposure to the glue may be indicated, as a conventional patch test may remain false-negative [211]. A patient from Japan, who apparently became sensitised by application to Dermabond^®^, later developed ACD after contact with an eyelash glue containing n-butyl cyanoacrylate and a household “super-glue” containing ethyl cyanoacrylate, which points to the possibility of cross-sensitivity to different cyanoacrylates [212], which has hitherto not much been studied.

In Belgium and Sweden, 15 patients presented with ACD caused by the FreeStyle^®^ Libre glucose sensor. All but 1 were patch tested with a baseline series, and with pieces and/or ultrasonic bath extracts of (the adhesive part of) the glucose sensor, to which all reacted. Isobornyl acrylate was patch tested, in various concentrations and vehicles, in 13 patients, and identified as culprit contact allergen in 12. Gas chromatography–mass spectrometry (GC-MS) of the sensors showed the presence of isobornyl acrylate in the sensor [213]. 

ACD caused by acrylic acid used in transcutaneous electrical nervous stimulation has been reported in an 81-year-old Dutch patient; the electrodes used, as well as some acrylates and acrylic acid 0.5% pet. caused strong to extreme positive patch test reactions [214]. An acrylate-free electrode was identified and could further be used by the patient. Fully cured polyurethane plastics are rarely a concern regarding sensitisation. However, in the case of a 23-year-old US female who was equipped with Invisalign™ clear dental aligners (made from polyurethane), both urticaria with angioedema and contact stomatitis developed, diagnosed as contact allergy by positive patch test reactions both to the aligner, and to diaminodiphenylmethane and hexamethylene diisocyanate [215]. 

Wearables can probably be regarded as “medical devices” in a broader sense—they may just as well cause contact allergic reactions, given the prolonged exposure, and liberation of potentially sensitising agents. This was observed in a 52-year-old woman who developed ACD 4 days after she had started to wear a new watch-like wearable fitness sensor; patch testing revealed a ++ reaction to methyl methacrylate and a + reaction to ethyl acrylate; prior use of acrylic nails (without adverse reaction reported) may have induced sensitisation in this case [216].

Modern dressings, also including stoma care products, but also dressings for chronic venous ulcers [217], may be a source of contact dermatitis. In case of stoma care products, localised dermatitis has often been considered to be due to bodily fluids or other irritation, however, according to a US study involving 18 such patients, contact dermatitis to adhesives and also to “shield” topical products is so frequent as to warrant systematic investigation and patch testing [218]. In a 10-year-old boy with epidermolysis bullosa simplex, patch testing was very challenging due to the vulnerability of his skin; however, by applying just a few contact allergens with a silicone dressing, contact allergy to cellulose gum (carboxymethylcellulose), an ingredient of the previously multiply used Urgotül^®^ dressing, could be diagnosed [219].

Suture materials have occasionally been reported as cause of partly severe “intracutaneous” ACD, as in the case of a 42-year-old woman who developed oedema and erythema 2 days after surgical removal of a tattoo. She had a +++ reaction to a suture tied to the skin for 48 h; the material was Poliglecaprone 25, a copolymer of glycolide and ε-caprolactone [220]. 

In patients with lower leg dermatitis, chronic venous insufficiency or chronic leg ulcers a high prevalence of contact sensitization is observed. A recent study of the IVDK covering 2003 to 2014 found that *Myroxylon pereirae* (balsam of Peru) (14.8% positive reactions), FM I (11.4%), lanolin alcohol (7.8%), colophonium (6.6%), neomycin sulfate (5.0%), cetearyl alcohol (4.4%), oil of turpentine (3.1%), and paraben mix (2.6%) are important contact allergens from the baseline series. Additional aimed testing often diagnoses other contact allergies, e.g., to topical antibiotics [221]. The gel of a catheter system used in a patient with a neo bladder contained sodium metabisulfite as preservative, which had caused ACD in the 68-year-old patient [222].

Also, implants of various types may cause ACD of skin covering the site, or symptoms of systemic allergic dermatitis. Nickel-release from a pacemaker, and contact allergy to nickel diagnosed by patch testing and a positive lymphocyte transformation test (LTT), had been the reason for a long history of futile revisions for presumed infection, until the diagnosis had finally been made [223]. In a follow-up study of 855 arthroplasty patients known to be sensitised to chromium, cobalt, nickel, or a cement component, 682 received their first implant, and the remainder a revision. Among the latter, 17 patients (2.0%) were revised because of allergic reactions. Allergic reactions were the cause for approximately 0.2% of all endoprosthetic revisions and for 9.8% of revisions in patients with sensitisation to one of the reviewed components. Potential contact allergens were strictly avoided in the replacement prosthesis, and outcome scores improved post-operatively [224].

### 3.14. Special Clinical Presentations

Contact allergic reactions may also be observed in the mucosa, e.g., the oral mucosa following exposure to (meth)acrylates, as in 2 British cases, after overnight application of a teeth whitening gel containing 2-HEMA and temporary fillings, respectively [225]. A Swedish patient suffering from long-standing cheilitis was found to be sensitised to the acacia honey he was fond of eating, as confirmed by patch testing which was also positive to propolis, yellow beeswax and *Myroxylon pereirae* [226]. A series of 91 patients with cheilitis were patch tested between 2001 and 2011 at the Mayo Clinic and were often found allergic to FM I, *Myroxylon pereirae* resin, dodecyl gallate, octyl gallate, and benzoic acid, leading to a final diagnosis of ACD in almost half of these patients [227]. 

A 32-year-old female with discoid cutaneous lupus erythematosus (DLE) developed, two weeks after a first episode of ACD secondary to hair dyeing, DLE lesions on the previously affected skin sites. This reaction pattern was repeated in the positive patch test sites to p-phenylenediamine and toluene-2,5-diamine, and immune-histologically confirmed as DLE. The authors point out that if patients present late, acute lesions may have subsided and a correct diagnosis of (additional) ACD will not be made [228]. Prurigo has not yet been linked with contact allergy; however, an analysis of IVDK data found that 35% of 639 patients with prurigo had positive patch test reactions [229], which may, in view of a lacking pattern, be independent of prurigo.

Apparent nail psoriasis in 2 Belgian patients was, in fact, due to contact allergy to formaldehyde contained in a nail hardener [230]. A 26-year-old woman presented with a quickly generalising erythema multiforme (EEM)-like rash spreading from her ankle, where she had applied propolis essence. Both the essence and propolis (10% pet.) confirmed contact allergy to this natural extract [231]. ACD caused by *Geranium robertianum* similarly caused an erythema multiforme-like reaction in a 76-year-old French pensioner, accompanied by mild fever (38 °C). A petal and a leaf, moistened with water, elicited a ++ reaction in patch testing [232]. 

Combined hand and foot dermatitis is often considered to be mainly of endogenous origin, however, according to a study comparing 125 patients with such combined eczema with 294 patients with hand eczema identified contact allergy in 51.8% of the patients, similar in both subgroups [233]. As a conclusion, these patients should be investigated for contact allergy just like hand dermatitis patients. 

Of 66 patients with itching and chronic vulvar complaints, use of topical botanical preparations was reported by 42. Of these, 14 had at least one relevant reaction, mainly to contact allergens in topical products and cosmetics, including 3 with relevant sensitisation to botanical products [234]. In a series of 124 Spanish patients with chronic perianal dermatitis (>4 weeks), systematic diagnostic work-up included patch testing. This most often revealed clinically relevant contact allergy to MCI/MI (*n* = 17), neomycin sulfate (*n* = 11), and caine mix (not specified; *n* = 6); the importance of testing own culprit products (in *n* = 20) was stressed [235]. 

### 3.15. General Aspects

Ageing is associated with alterations in T-cell-mediated immune function. The overall incidence of delayed-type cutaneous hypersensitivity increases with age. The patch test records of all patients (45,110) tested with a modified European baseline series between 1985 and 2014 at St John’s Institute of Dermatology, London were reviewed [236]. The data show that the age distribution of patients presenting for patch testing increases until the early 20s and then decreases thereafter with the pattern being consistent across 3 decades of testing. The overall prevalence of patients sensitized to at least 1 contact allergen increases throughout the lifespan with the rate of increase being most rapid before the age of 20 years, with peak incidence in individuals of age 40 to 60 years and a modest decline thereafter. Patients reacting to 1 or 2 contact allergens exhibit an increase in prevalence until the age of 20 years followed by a plateau. Those reacting to 3 or more contact allergens show a steady increase throughout the lifespan.

The reproducibility of patch testing has been the subject of several studies. Recent evidence from a small study with 31 subjects, 19 allergic to gold and 12 to nickel, showed a good reproducibility of serial dilution tests on different areas on the back, even if the strength of the positive reactions varied [237]. Patch testing on skin with part of the epidermis removed by prior standardised tape stripping (“strip patch testing”) has been advocated by some to increase the sensitivity of the patch test. This method has now been shown to be as reliable (reproducible upon synchronous application) as conventional patch testing [238]. However, it has been pointed out that a similar increase in sensitivity can also be achieved by increasing the patch test contact allergen dose and using the standard patch test procedure, if a false-negative result with the standard dose is suspected. 

A major determinant of patch test reproducibility is the degree of standardisation of patch testing, very importantly including the dose/area applied [1]. The use of micropipettes to deliver liquid contact allergens has, in the meantime, been accepted to ensure sufficient precision; in the case of pet-based substances, i.e., the vast majority of contact allergens, similar efforts of reliable dosing need to be made, as variability between users of investigator-loaded chambers is large and can be reduced by the use of suitable dispensers [239]. 

If clinically relevant contact allergy has been identified by patch testing, firm and lasting knowledge of the patient on the contact allergen(s) diagnosed is extremely important for successful secondary prevention and a good health-related quality of life [240]. Two years after a patch test, 149 of 199 invited patients responded to a follow-up study addressing their recollection of patch test results—which was found to be quite good: only 13% did not remember their occupational contact allergy [241]. The need to patch test patients with occupational hand dermatitis avoiding overly long delay is underlined by the (repeated) observation of a high share of potentially relevant positive patch test reactions, e.g., as observed in Danish healthcare workers [242]. An even longer follow-up of 10 years after patch testing of 256 Swedish patients revealed a decrease of health-related quality over time [240] which may partly be explained by forgetting such relevant information. A practically useful approach to diagnosing sources of exposure and sensitisation which are not obvious on first sight is demonstrated by E. Özkaya, illustrated by 2 cases in which positive patch test reactions to pieces of used shoes and bandages, respectively, pointed to previously unrecognised contamination with topical NSAID creams, and sensitisation to these [243]. 

## 4. Conclusions

Several new contact allergens are identified every new year, constantly expanding the horizon of known causes of ACD. Furthermore, new uses of well-known contact allergens may lead to new exposures and thus sensitisation risks. Timely recognition through the invaluable tool of investigative patch testing, if necessary, combined with analytical chemistry and reporting helps to generally improve diagnostic accuracy; the present review is intended to contribute to this goal by briefly summarising new findings in the past two years.

## Figures and Tables

**Table 1:** Case reports of new contact allergens identified in terms of biocides (preservatives, but also including herbicides and pesticides) other than isothiazolinones. F, female, M, male; age in parentheses. NT, not tested. Patch tests usually read on day (D)3, D4, or D7.

Substance	Patients	Clinical Problem	Patch Test	Comment	Ref.
Benzoic acid, Sodium benzoate	M (12)	Cheilitis caused by Coca Cola and canned beans	Sodium benzoate 5% pet: ++, benzoic acid 2%: +		[244]
Didecyldimethylammonium chloride	12	Dermatitis	0.1% pet.: 2 relevant pos.	Several false-positive; test dilution series	[245]
Pethoxamid	M (49) production worker	Acute dermatitis after accidental exposure	0.01% +, 0.03–1% +++	Agricultural workers may be at risk, too	[246]
Polyhexamethylene biguanide	F (59)	Chronic leg ulcer, multiple sensitization, dermatitis after wound gel	PHMB 5% aq. ++		[247]
Polyhexamethylene biguanide	F (46)	Hand dermatitis after contact lens cleaning solution	PHMB 5% aq. +	Found In 16/35 contact lens solutions	[248]
Polyhexamethylene biguanide	M (13)	Peristomal dermatitis due to foam dressing	PHMB 5% aq. +	Also allergic to alkyl glucosides	[249]
1-Propanol	F (56) nurse	Hand dermatitis after Sterillium^®^	1-propanol 10% aq.: +	2-Propanol: neg.	[250]
Sodium metabisulfite	357/12,156	Various products, e.g., ophthalmic	1% pet.	40% clinically relevant	[251]

**Table 2:** Case reports of new contact allergens identified in pharmaceutical agents following skin exposure. ACD, allergic contact dermatitis; F, female, M, male; age in parentheses. LTT, lymphocyte transformation test; NT, not tested; TTS, transdermal therapeutic system. Patch tests usually read on day (D)3, D4, or D7.

Substance	Patients	Clinical Problem	Patch Test	Comment	Ref.
*N*-Acetylcysteine	1 F (32) nurse	Hands, arms, face dermatitis	10% pet: +	LTT also positive	[252]
ϵ-Aminocaproic acid	1 F (78) Japanese	Periorbital ACD due to sodium hyaluronate ophthalmic solution	1 and 2% aq.: +		[253]
Brimonidine tartrate	2 M (50,60)	Face dermatitis after Mirvaso^®^ gel	Gel “as is”: neg. resp. ^(1)^	ROAT pos. too	[254]
Camphorquinone	1 M (30)	Contact stomatitis after periodontal dressing	1% pet.: +++		[255]
Chlorhexidine	82/8497 pos.	Antiseptics > cosmetics	2 salts used. ^(2)^	Accidental, symptomatic re-exposure	[256]
Codeine (and naloxone)	2 F (38,58)	ACD in pharmaceutical workers	5% pet.; naloxone 10% pet.: +, +	No reactions to synthetic opiates	[257]
Efinaconazole	1 M (74)	Toe dermatitis after antifungal solution	Solution “as is”: ++; 10% pet.: +		[258]
Ketotifen (eyedrops)	1 F (32)	3 years chronic conjunctivitis	0.7% aq. and 2.5% pet.: + and ++		[259]
Methyl aminolaevulinate	1 F (53) KID syndrome	Local and systemic dermatitis after PDT	Metvixia^®^ “as is”: ++, MAL 21% pet. +		[260]
Olaflur	1 F (35)	Cheilitis for 9 months	Toothpaste 1% aq., ++, olaflur ++	Olaflur concentration not disclosed	[261]
Ozonated olive oil	2 F (23)	(a) Cheilitis (b) foot dermatitis	(a) neg. (b) ++ to product	Irritant reactions in controls	[262]
Polymyxin B	18/795 pos.	12/18 past exposure	Polymyxin B sulfate 3% pet.	2.3% pos. in consecutive pat.	[263]
Rotigotine	1 (69) with Parkinson’s dis.	ACD under TTS	10% pet. ++	Placebo TTS neg. in ROAT	[264]
Sirolimus	1 F (32)	Localised ACD	Rapamune^®^ “as is” and 1:3 aq.: +++/+	After after laser treatment of port wine stain	[265]
Tea tree oil	1 F (32)	ACD after applying burn gel	Multiple extracts, incl. *Melaleuca alternifolia* 5% pet.: ++	Burns induce barrier dysfunction, increasing risk	[266]
Thebaine (and oripavine)	1 M (35)	ACD in pharmaceutical worker	5% aq.: ++ and +, resp.	Morphine and noscarpine neg.	[267]
Tobramycin	2 F (35,59), 1 M (66)	Eyelid dermatitis after ophthalmic ointments	20% pet.: + to ++	Not detected by neomycin	[268]
Turmeric (*Curcuma longa*)	1 F (60)	Localised dermatitis after massage oil	0.01% wt/wt pet. ++	Also common spice and dye	[269]

^(1)^ Only early readings positive (+ and ++, resp.), late main reading negative; brimonidine tartrate 0.5% aq. negative. ^(2)^ Both chlorhexidine diacetate and chlorhexidine digluconate tested, first half of study period 1% aq., second half 0.5% aq.

**Table 3:** Case reports dimethylaminopropylamine; F, female, of new contact allergens identified in cosmetic products. ACD, allergic contact dermatitis; DMAPA, M, male; age in parentheses. NT, not tested; ROAT, repeated open application test. Patch tests usually read on day (D)3, D4, or D7.

Substance	Patients	Clinical Problem	Patch Test	Comment	Ref.
2-Amino-4-hydroxyethylaminoanisole sulfate	F (68) + F (66) and 11 further patients	Severe scalp dermatitis after hair dyeing	2% pet. +++	Coupling agent	[270]
Arbutin	1 F (60) Japanese	Face dermatitis from skin whitening cream	Cream “as is” and arbutin. 3% aq.: ++		[271]
Cetearyl isononanoate	1 M (2)	Acute dermatitis after sunscreen application	4% pet.: +++	Third reported case	[272]
Cocamide diethanolamine (DEA)	18/1767 pos.	Hand dermatitis, shampoo/liquid soap	0.5% pet.	Frequent use, contact allergy relatively rare	[273]
Cocoamphopropionate	5 F (20–34)	Hand dermatitis from hand cleanser	1% pet.	Aminoethylethanolamine possible culprit	[274]
Cocamidopropylamine oxide	F (33)	Chronic hand dermatitis	ROAT 1% aq. pos. day 3	Irritating when patch tested; DMAPA as possible culprit	[275]
3-*o*-Ethyl-l-ascorbic acid	F (37)	Face dermatitis after skin-lightening cream	10% aq.: +		[276]
Ethylhexylglycerin	F (50)	Widespread dermatitis	5% pet. ++	In 2 skin care products	[277]
Ethylhexylglycerin	12 F (29–81), 1 M (79)	Face, hand and axillary dermatitis	0.3–10% pet.	“low-risk, but highly relevant”	[278]
*Glycyrrhiza inflata* extract	2 M (60, 70)	Face dermatitis due to aftershave	1% pet./ethanol: ++/+		[279]
Hydroxypropyl tetrahydropyrantriol	F (65)	Eyelid dermatitis after “anti-ageing” cream	9% alc./aq: ++	Is Pro-Xylane™	[280]
Liquorice flavonoids	F (39) Japanese	Face dermatitis from skin whitening cream	Product “as is” +, liquorice flavonoid 2% pet. +	LC-MS analysis: licoflavone A and glabridin	[281]
*Magnolia officinalis* bark extract	F (66)	Face dermatitis from “anti-ageing” cream	0.5% pet.: +		[282]
Panthenyl ethyl ether	F (65)	ACD to emollient stick	Product “as is” ++, 30% pet. ++	ROAT pos. after 2 days	[283]
Pentaerythrityl tetracaprylate/tetracaprate	F (36)	Face dermatitis after night cream	5% pet. ++		[284]
PEG-22/dodecyl glycol copolymer	F (45)	ACD due to Cicalfate^®^ cream	20% pet.: ++	cream “as is” PT neg., but ROAT pos.	[285]
Phenylethyl resorcinol	2 F (30,50)	Face dermatitis after sunscreen	2% pet.: +++	Strong skin-lightening agent	[286]
*Scutellaria baicalensis* extract	F (49)	Face dermatitis after Resveratrol BE cream^®^	0.5% aq.: +		[287]
Steareth-10	F (36)	ACD due to wet wipes	5% aq.: +	Traces of stearyl alcohol as cause?	[288]
Thioctic acid	F (31)	Face dermatitis from anti-wrinkle cream	5% pet. ++	Also a dietary supplement	[289]
Thioctic acid	F (64)	Periorbital dermatitis from eye drops	3% aq.: ++		[290]
Vitamin K1 oxide	M (6), F (35)	Erythema multiforme-like reaction	5% pet.: +, +++	syn.: phytomenadione epoxide	[291]
